# 

**Published:** 1877-08

**Authors:** 


					﻿Vol. 24.
AUGUST, 1877.
No. 8.
TWENTY-FOURTH YEAR.
HALL’S
Journal of Health.
PUBLISHED AT BIBLE HOUSE,
THIRD AVENUE, CORNER OF NINTH STREET,
NEW YORK.
E. H. GIBBS, M.D., Editor.
AGENTS FOE THE TRADE:
AMERICAN NEWS COMPANY, 121 NASSAU STREET.
NEW YORK NEWS COMPANY, 18 BEEKMAN STREET.
Terms, $1.50 a Year.
SINGLE NUMBER, 15 CENTS.
Kent & Co., Printers, 11 Frankfort St., New York.
				

